# Anticancer activity of caffeic acid n-butyl ester against A431 skin carcinoma cell line occurs via induction of apoptosis and inhibition of the mTOR/PI3K/AKT signaling pathway

**DOI:** 10.3892/mmr.2021.12011

**Published:** 2021-03-16

**Authors:** Ning Zeng, Tang Hongbo, Yi Xu, Min Wu, Yiping Wu

Mol Med Rep 17: 5652-5657, 2018; DOI: 10.3892/mmr.2018.8599

An interested reader drew to our attention that, in the above paper, several of the figures appeared to contain strikingly similar data to those published in other articles by different authors from different research institutions, including (as examples) Fig. 8 (cf. Fig. 8 in Fang, K *et al*: ‘Antiproliferative effects of matricine in gemcitabine-resistant human pancreatic carcinoma cells are mediated via mitochondrial-mediated apoptosis, inhibition of cell migration, invasion suppression, and mammalian target of rapamycin (mTOR)-TOR/PI3K/AKT signalling pathway’, Med Sci Monit 25: 2943-2949, 2019), Fig. 4 (cf. Fig. 7 in He, W *et al*: ‘Arglabin is a plant sesquiterpene lactone that exerts potent anticancer effects on human oral squamous cancer cells via mitochondrial apoptosis and downregulation of the mTOR/PI3K/Akt signaling pathway to inhibit tumor growth *in vivo*’, J BUON 23: 1679-1685, 2018) and Fig. 7 (cf. Fig. 7B in Yu, Y *et al*: ‘Globularifolin exerts anticancer effects on glioma U87 cells through inhibition of Akt/mTOR and MEK/ERK signaling pathways *in vitro* and inhibits tumor growth *in vivo*’, Biochemie 42: 144-151, 2017). The authors also independently informed the Editorial Office that they were unable to repeat the experiments shown in Fig. 4, and requested a retraction.

Given the overall problems that have come to light with this paper, the Editor of *Molecular Medicine Reports* has agreed with the authors that this article should be retracted from the publication. The Editor and the authors apologize for any inconvenience caused.

